# An Acousto-Optical Sensor with High Angular Resolution

**DOI:** 10.3390/s120303739

**Published:** 2012-03-20

**Authors:** Gennady Kaloshin, Igor Lukin

**Affiliations:** V.E. Zuev Institute of Atmospheric Optics SB RAS, Tomsk, 634021, Russia

**Keywords:** acousto-optical sensor, atmospheric turbulence, interference, laser beam

## Abstract

The paper introduces a new laser interferometry-based sensor for diagnosis of random media by means of high accuracy angle measurements and describes the results of its development and testing. Theoretical calculations of the dependence of the range of the laser interferometer on laser beam parameters, device geometry, and atmospheric turbulence characteristics are reported. It is demonstrated that at moderate turbulence intensities corresponding to those observed most frequently in turbulent atmosphere at moderate latitudes and with low interference contrast values, the performance range of the laser interferometer-based device exceeds 5 km.

## 1. Introduction

The current state-of-the-art of acousto-optic device development allows for their wide application, particularly in diagnosis of random media (by random media we understand media with fluctuations of the refraction index) by means of high accuracy angle measurements [1–3]. These may be applied in the control of slowly changing transient processes in turbulent atmosphere, gaseous and liquid media. In particular, they may be valuable as sensing instruments in atmospheric optics for the study of intensity fluctuations during the propagation of optical radiation and as applied to sensing the Earth, optical refraction spectroscopy, photothermal deflection spectroscopy for the control of transient processes in gaseous and liquid media, in investigation of mirage detection and reflectance techniques, in geodesy, optical profilometry and ranging.

Modern photoacoustic grating deflectors (PAD) are characterized by high resolution and fast response. Along with the simple design, ease of control, low power consumption, and small size, these advantages have aroused interest in using PADs for the design of atmospheric laser interferometers. A fundamental problem here lies in ensuring high precision in the measurement of angles, which may be, at least, 5,000 times smaller than 1 second of arc.

In [4,5] the authors introduce a method in which during synchronous scanning of two laser beams in the region of their superposition, wave interference is observed, and the frequency of the resultant oscillation is uniquely associated with the source direction. It is known that the possibility of registering interference contrast is determined by the presence of random non-uniformities in the permittivity of air on the path. Therefore, it appears interesting to estimate an operation range of the method, depending on turbulence characteristics and optical scheme parameters of the device.

## 2. Interferometric Sensor of Specifying Direction

**Figure 1.**
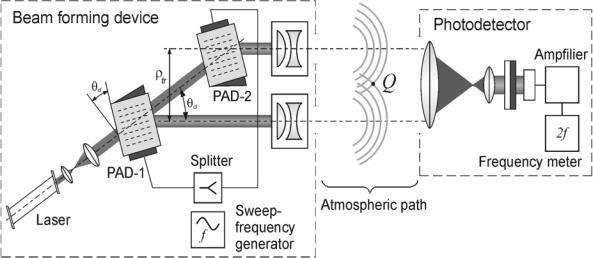
The schematic of the atmospheric laser sensor.

An optical schematic of the atmosphere laser path interferometer (laser sensor–LS) employing this method for a one-dimensional case is shown in Figure 1 (for a two-dimensional case, a laser beam is scanned across two coordinates using either two one-dimensional PADs in tandem or one PAD cell, in which two orthogonal acoustic waves are excited).

A laser beam passes sequentially through two acoustic cells, PAD-1 and PAD-2, whose medium density is varied under the influence of ultrasound waves from a sweep-frequency generator. The acoustic waves produce a phase grating on which laser radiation is diffracted. With the help of collimators of a beam forming device, further referred to as BFD, and diffracted beams are directed towards the atmosphere path, where they produce an interference pattern in the region of mixing. Characteristics of the optical system and positioning of the optical elements are chosen so that polarization of both beams and their intensities are similar. A photodetector registers and separates the received signal frequency, which uniquely correlates with direction to BFD.

Position of the laser beams in space is determined by the relation sin(θ*_d_*) = λ*f_a_*/ν*_a_*, where θ*_d_* is the diffraction (scanning) angle; λ is the optical radiation wavelength in vacuum; *f_a_* is the acoustic wave frequency; ν*_a_* is the acoustic wave speed in a PAD. At small diffraction angles, this may be transformed to θ*_d_* ≅ λ*f_a_*/ν*_a_*. For a He–Ne laser (λ = 0.63 μm) and acoustic wave frequency *f_a_* = 10 × 10^6^ Hz, θ*_d_* ≅ 2°. If θ*_d_* is increased 10-fold by means of optical amplification, the diffraction angle of 1 second of arc corresponds to the frequency of 5.5 kHz. Hence, setting direction to within 1 second of arc is not technically challenging. As such, the accuracy of measurements may exceed at least 1/5,000 seconds of arc. This unveils wide opportunities in application of the method both in research and engineering. In particular, it may be valuable in optical refraction spectroscopy, photothermal deflection spectroscopy for the control of slowly changing transient processes in gaseous and liquid media and plasma, in atmospheric optics for the study of intensity fluctuations during the propagation of optical radiation, in investigation of mirage detection and reflectance techniques, in geodesy, optical profilometry, ranging, and navigational course detection. Now for control of slowly changing transient processes laser remote techniques may be used, thus the possibilities of these methods are limited by the sizes of laser beams. The LS method offers essentially the best resolution possibilities as in it the resolution is defined by the width of an interference band.

## 3. Distortions of an Interference Pattern Formed by a PAD

Various aspects of interference pattern formation in turbulent atmosphere have been studied in [6–8]. For the purpose of this paper, we summarize a theoretical approach described in [7,8] applying it to a more realistic model of optical radiation sources.

Let us consider the following model of a BFD and a photodetector: coherent radiation sources located at the points {0, **ρ**_1_} and {0, **ρ**_2_}, respectively, emit laser beams parallel to each other and the *OX*-axis in the direction of positive *x* growth; the photodetector is located at point *Q* (see Figure 1) with coordinates {*x*, **ρ**}. Vector **ρ***_tr_* = (**ρ**_2_ – **ρ**_1_) denotes spacing between the radiation sources producing the interference pattern.

Let the optical radiation from each source be represented as a partially coherent Gaussian beam with initial amplitude *U_0_*, initial radius *a*_0_, radius of the wave front curvature in the center of radiating aperture *R*_0_, and initial coherence radius ρ*_k_*. An assumption about complete identity of the sources somewhat simplifies the problem without introducing serious restrictions. The photodetector is represented as a quadratic detector, which reacts to intensity of arriving radiation, and its signal is proportional to instantaneous interference pattern intensity at the detector location, which can be written as:

(1) $$I(x,\rho )=U_{1}(x,\rho )\;U_{1}^{*}(x,\rho )+U_{2}(x,\rho )\;U_{2}^{*}(x,\rho )+2\;ℜe[U_{1}(x,\rho )\;U_{2}^{*}(x,\rho )]$$

where *U_j_*(*x*,**ρ**) is the optical wave field of one source; *j* = 1, 2. In the following we consider that **ρ**_1_ = –**ρ***_tr_*/2 and **ρ**_2_ = **ρ***_tr_*/2.

For the above-formulated boundary conditions, an equation describing the function of mutual second-order field coherence for two Gaussian optical radiation beams has the following solution when using quadratic approximation of the structural function of fluctuations of a complex optical wave phase:

(2) $$\begin{array}{lll} \langle U_{j}(x,\rho )\;U_{j^{\prime }}^{*}(x,\rho )\rangle & = & \frac{U_{0}^{2}\;a_{0}^{2}}{a^{2}(x)}\text{exp}[-\frac{{\left(\rho -\rho_{j}\right)}^{2}}{{2a}^{2}(x)}-\frac{{\left(\rho -\rho_{j^{\prime }}\right)}^{2}}{{2a}^{2}(x)}-i\frac{\delta (x)}{a^{2}(x)}(\rho_{j}-\rho_{j^{\prime }})\rho +\\ & & +i\frac{\delta (x)}{{2a}^{2}(x)}(\rho_{j}^{2}-\rho_{j^{\prime }}^{2})-\frac{{\left(\rho_{j}-\rho_{j^{\prime }}\right)}^{2}}{\rho_{c}^{2}(x)}] \end{array}$$

where $a(x)=a_{0}{\left[{\left(1-\mu \right)}^{2}+\Omega_{0}^{-2}(1+a_{0}^{2}\rho_{k}^{-2}+(4/3)a_{0}^{2}\rho_{0}^{-2})\right]}^{1/2}$ is the mean radius of the optical radiation beam; $\delta (x)=\Omega_{0}[-\mu (1-\mu )+\Omega_{0}^{-2}]$ is a geometrical factor related to mutual curvature of the mean wave fronts of the beams; $\rho_{c}(x)=\sqrt{3}\rho_{0}{\left[\frac{{\left(1-\mu \right)}^{2}+\Omega_{0}^{-2}(1+a_{0}^{2}\rho_{k}^{-2}+(4/3)a_{0}^{2}\rho_{0}^{-2})}{\left(\mu^{2}+\Omega_{0}^{-2})(1+(3/4)\rho_{0}^{2}\rho_{k}^{-2}\right)}\right]}^{1/2}$ is the radius of mutual coherence of the two optical beams; μ = *x*/*R*_0_ is the focusing parameter; Ω_0_ = *ka*_0_^2^/*x* is the Fresnel number of the radiating aperture; *k* = 2π/λ is the optical wave number; ρ_0_ = (0.3642*C*_ε_^2^*k*^2^*x*)^3/5^ is a coherence radius of a plane optical wave in turbulent atmosphere; *C*_ε_^2^ is a structural fluctuations parameter of dielectric permittivity in turbulent atmosphere; *j*, *j*′ = 1, 2. Using Equations (1) and (2), it is possible to obtain an expression for mean intensity of the interference pattern produced by two partially coherent laser beams in turbulent atmosphere. The latter allows specifying simple and intuitive conditions for restricting the choices of laser beam parameters and BFD schemes using standard characteristic values for atmospheric turbulence above the sea surface [7,8].

If *a*(*x*) >> ρ*_tr_*, linear dimensions *l*_int_(*x*) of the region of formation of the interference pattern are described using a value approximately equaling the laser beam diameter:

(3) $$l_{\text{int}}\;\left(x\right)≅2a(x).$$

The interference band maxima are located at points **ρ**_max_, while the minima are located at points **ρ**_min_. For simplicity, let us assume that **ρ**∣∣**ρ***_tr_*. Then, interference band width Δ*l*_int_(*x*) can be assessed using equation:

(4) $$\Delta l_{\text{int}}\;(x)=2\left|\rho_{\text{max}}-\rho_{\text{min}}\right|=2\pi a^{2}(x)/\delta (x)\rho_{\mathrm{tr}}.$$

At the same time, visibility of a distorted interference pattern obtained using average intensity when **ρ**_max_ ≈ **ρ**_min_ ≈ **ρ**, is equal to:

(5) $$\nu (x)=\frac{\langle I(x,\rho_{\text{max}})\rangle -\langle I(x,\rho_{\text{min}})\rangle }{\langle I(x,\rho_{\text{max}})\rangle +\langle I(x,\rho_{\text{min}})\rangle }≅\{1/\text{cosh}[\rho_{\mathrm{tr}}\cdot \rho /a^{2}(x)]\}\text{exp}[-\rho_{\mathrm{tr}}^{2}/\rho_{c}^{2}(x)].$$

It is evident that the method works only if there is at least one complete interference band in the interference image field; therefore, the following necessary condition follows from Equations (3) and (4):

(6) $$\rho_{\mathrm{tr}}\geq \pi a(x)/\delta (x).$$

Additionally, the visibility of the middle interference pattern is satisfactory as long as the coherence radius of the registered optical field exceeds the magnitude of transverse displacement of the beams at the point of observation. Hence, when considering visibility near the centre of the interference pattern, by simplifying Equation (5) one obtains:

(7) $$\rho_{\mathrm{tr}}\leq \sqrt{-\text{ln}[\nu (x)]}\;\rho_{c}(x)$$

By combining Equation (6) and (7), the following equation is derived:

(8) $$\pi a(x)/\delta (x)\leq \rho_{\mathrm{tr}}\leq \sqrt{-\text{ln}[\nu (x)]}\;\rho_{c}(x).$$

In order to fulfill Equation (8), the right part must at least exceed the left part; otherwise (in the case of collimated beams μ = 0) this is equivalent to a requirement to limit the initial size of the laser beams *a*_0_ to obey the condition:

(9) $$a_{0}\leq (\sqrt{-\text{ln}[\nu (x)]}/\pi )\;{\left[1/(3\rho_{0}^{2})+1/(4\rho_{k}^{2})\right]}^{-1/2}.$$

It must be noted that for horizontal atmospheric paths not longer than 10 km, Equation (9) imposes quite severe limitations on the laser beam parameters, which may only be satisfied for sufficiently narrow and highly coherent laser beams.

## 4. Choice of Parameters for the LS Optical Scheme

**Figure 2.**
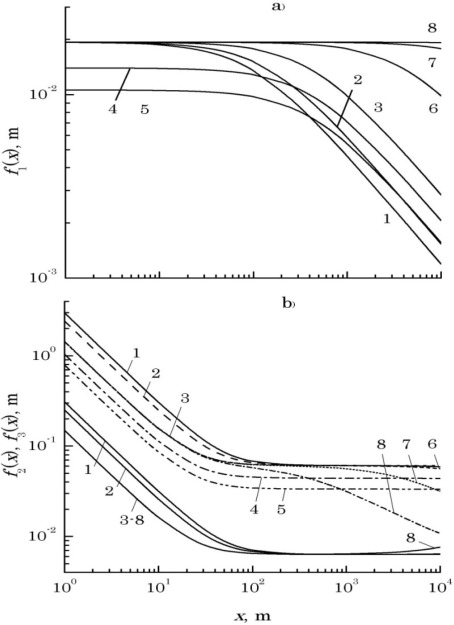
Nomograms used for the selection of initial values of laser beam parameters and spacing of their optical axes: function *f*_1_(*x*) for a choice of initial values of beams radiuses: curves 1–8 (**a**); functions *f*_2_(*x*) and *f*_3_(*x*) for a choice of size of spatial diversion their optical axes (**b**): *f*_2_(*x*)–the bottom group of solid curves 1–8; *f*_3_(*x*)–the top group of curves of different linestyles 1–8.

Figure 2(a) shows plots of the function:

$$f_{1}(x)=\frac{\sqrt{-\text{ln}[\nu (x)]}}{\pi }\;{\left[\frac{\rho_{0}^{-2}}{3}+\frac{\rho_{k}^{-2}}{4}\right]}^{-1/2},$$

for different values of parameters of the problem. Curves *1*, *2*, and *3* demonstrate the influence of optical radiation wavelength λ on function *f*_1_(*x*): *1* – λ = 0.51, *2* – 0.63, and *3* – 1.06 μm (other parameters are as follows: ρ*_k_* = 2 cm, *v*(*x*) = 0.1, *C*_ε_^2^ = 10^−13^ m^−2/3^); curves *3*, *4*, and *5* demonstrate sensitivity of *f*_1_(*x*) to the contrast of the interference pattern *v*(*x*): *3* – *v*(*x*) = 0.1, *4* – 0.3, *5* – 0.5 (in this case, λ = 1.06 μm, ρ*_k_* = 2 cm, *C*_ε_^2^ = 10^−13^ m^−2/3^), while curves *3*, *6*, *7*, and *8* show the dependence of *f*_1_(*x*) on the level of dielectric permittivity fluctuations in turbulent atmosphere *C*_ε_^2^: *3* – *C*_ε_^2^ = 10^−13^ m^−2/3^, *6* – 10^−14^, *7* – 10^−15^, *8* – 10^−16^ m^−2/3^ (λ = 1.06 μm, ρ*_k_* = 2 cm, *v*(*x*) = 0.1). It appears that in order for condition (9) to be satisfied with the path length range 1 m to 10 km, the initial radii of the laser beams *a*_0_ must not exceed 1…2 mm.

At known parameters of the optical beams, Equation (8) allows choosing the distance ρ*_tr_* between the radiation sources, producing the interference pattern. Figure 2(b) demonstrates plots of the functions:

$$f_{2}(x)=\pi \frac{{\mathrm{ka}}_{0}^{3}}{x}{\left[1+x^{2}k^{-2}a_{0}^{-4}(1+a_{0}^{2}\rho_{k}^{-2}+(4/3)a_{0}^{2}\rho_{0}^{-2})\right]}^{1/2}$$

(solid lines) and:

$$f_{3}(x)=\frac{{\mathrm{ka}}_{0}^{2}}{x}\sqrt{-\text{ln}[\nu (x)]}{\left[\frac{1+x^{2}k^{-2}a_{0}^{-4}(1+a_{0}^{2}\rho_{k}^{-2}+(4/3)a_{0}^{2}\rho_{0}^{-2})}{\left(\rho_{0}^{-2}/3)+(\rho_{k}^{-2}/4\right)}\right]}^{1/2}$$

(dashed lines) calculated for different values of the parameters of the problem.

Curves *1*, *2*, and *3* indicate the influence of λ on *f*_2_(*x*) and *f*_3_(*x*): *1* – λ = 0.51, *2* – 0.63, and *3* – 1.06 μm (in this case, *a*_0_ = 2 mm, ρ*_k_* = 2 cm, *v*(*x*) = 0.1, *C*_ε_^2^ = 10^−16^ m^−2/3^), curves *3*, *4*, and *5* are calculated, respectively, for the following values of the interference contrast *v*(*x*): 0.1 (*3*), 0.3 (*4*), and 0.5 (*5*) (other parameters are constant: λ = 1.06 μm, *a*_0_ = 2 mm, ρ*_k_* = 2 cm, *C*_ε_^2^ = 10^−16^ m^−2/3^), while curves *3*, *6*, *7*, and *8* are calculated for different levels of dielectric permittivity fluctuations of turbulent atmosphere *C*_ε_^2^: 10^−16^ m^−2/3^ (*3*), 10^−15^ m^−2/3^ (*6*), 10^−14^ m^−2/3^ (*7*), and 10^−13^ m^−2/3^ (*8*) (λ = 1.06 μm, *a*_0_ = 2 mm, ρ*_k_* = 2 cm, *v*(*x*) = 0.1). Since the spacing ρ*_tr_* between the radiation sources producing the interference pattern, must exceed the value of *f*_2_(*x*) and be below the value of *f*_3_(*x*), it is possible to estimate acceptable values of ρ_*tr*_ within the 1–5 cm range from Figure 2(b) for path lengths from 20 m to 10 km.

## 5. Assessment of the LS Ranging

As far as the major limitation for the range of action of a LS in turbulent atmosphere is related to decreasing visibility of the interference pattern, in accordance with Equation (7), the required estimate can be obtained by solving a nonlinear equation of the form:

(10) $$x=f_{4}(x),$$

in which:

$$f_{4}(x)=\frac{{\mathrm{ka}}_{0}^{2}\sqrt{-\text{ln}[\nu (x)]}}{\rho_{\mathrm{tr}}}\sqrt{\frac{1+x^{2}k^{-2}a_{0}^{-4}(1+a_{0}^{2}\rho_{k}^{-2}+(4/3)a_{0}^{2}\rho_{0}^{-2})}{\left(\rho_{0}^{-2}/3)+(\rho_{k}^{-2}/4\right)}}.$$

**Figure 3.**
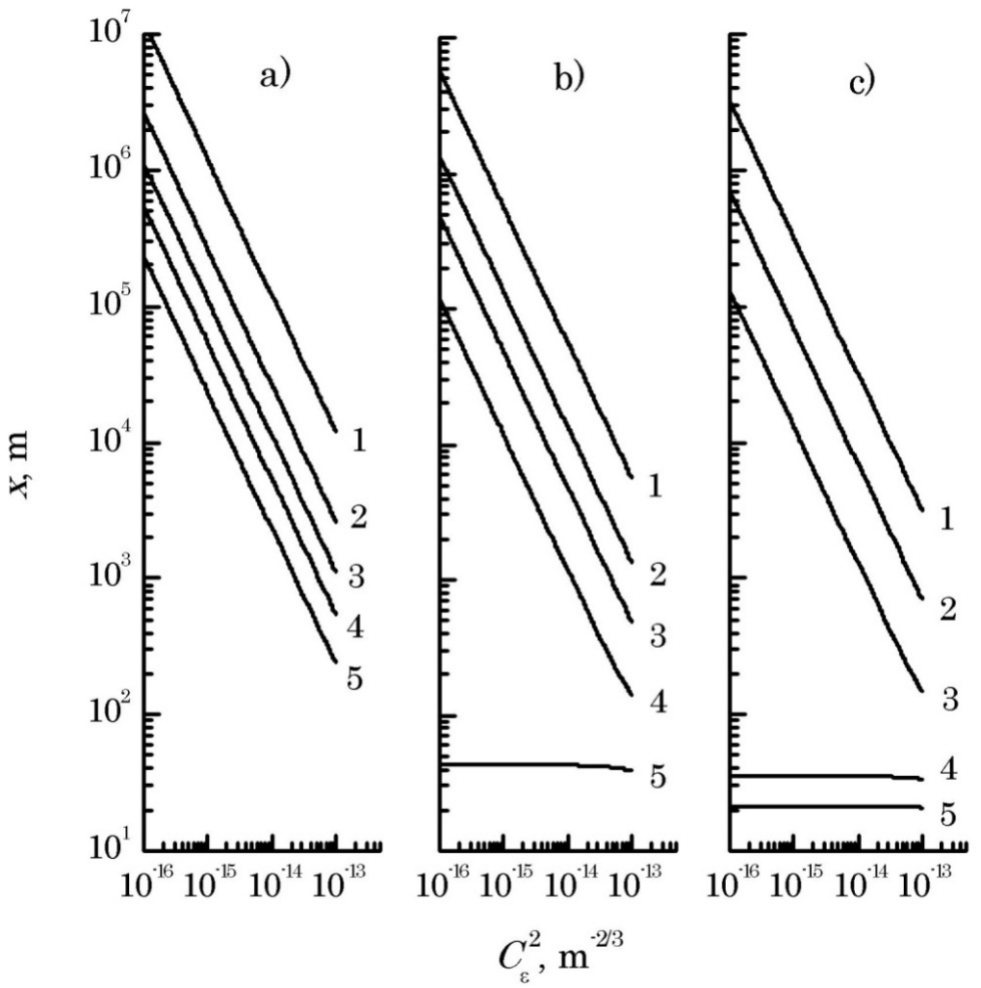
Laser beam range as a function of the structural parameter of atmospheric turbulence at radiation wavelength λ = 1.06 μm and interference pattern visibility v(x) = 0.1 (**a**), 0.3 (**b**) and 0.5 (**c**) for different distances between the beam centers ρtr = 0.01 (1), 0.02 (2), 0.03 (3), 0.04 (4), and 0.05 m (5).

Equation (10) has been solved by the simple iterations method. Results of the estimates of the range of action of a LS are presented in Figure 3 for all values of the structural parameter of air permittivity fluctuations, valid for the near-water atmospheric layer [7,8], and for three values of interference pattern visibility and optical radiation wavelength λ = 1.06 μm. It is assumed that the laser radiation propagated along a horizontal path at the height of 10…20 m above the water surface.

## 6. Conclusions

Based on the results presented in this work, the following conclusions can be drawn:

1. A decrease in the distance between the optical radiation sources results in lowering of the contrast value of the registered interference pattern; while an increase in the radiation wavelength results in a growth in the range of BFD.
2. At a lower value of the optical wavelengths interference pattern contrast (*v*(*x*) = 0.1), the device ranging exceeds 5 km, for the source spacing of 1 to 5 cm, and the mean turbulence intensity of 10^−15^ m^−2/3^ which is most commonly observed in the turbulent atmosphere at moderate latitudes.
3. At higher interference pattern contrast values, in order for the laser interferometer to operate efficiently the spacing ρ*_tr_* of radiation sources producing the interference pattern reduces (ranging from 1 to 4 cm for *v*(*x*) = 0.3, and from 1 to 3 cm at *v*(*x*) = 0.5).

In summary, this paper describes the operation of an atmospheric laser path interferometer based on the mean intensity analysis of a PAD-formed interference pattern used for specifying interference of BFD parameters, thereby providing the required range of operation for the LS above the ground surface for all possible values of turbulence parameters in weakly turbid atmosphere.

This unveils wide opportunities for application of the method both in research and engineering. In particular, it may be valuable in optical refraction spectroscopy, photothermal deflection spectroscopy for the control of transient processes in gaseous and liquid media and plasma, in atmospheric optics for the study of intensity fluctuations during the propagation of optical radiation, in investigation of mirage detection and reflectance techniques, in geodesy, optical profilometry, ranging, and navigational course detection.
